# Effect of the AADE7 Self-Care Behaviors Framework on Diabetes Education Management in a Shared Care Model

**DOI:** 10.1155/2024/7278207

**Published:** 2024-03-01

**Authors:** Yunxia Liu, Chenhui Liu

**Affiliations:** ^1^Beijing Ruijing Diabetes Hospital, Beijing 100079, China; ^2^Chengdu Ruien Diabetes Hospital, Chengdu 610017, China

## Abstract

**Background:**

Diabetes self-management education (DSME) provides diabetic patients with knowledge of diabetes, requires attention and recording of dietary habits, and increases the frequency and accuracy of blood glucose monitoring. DSME also achieves better blood glucose control, thus benefiting diabetic patients and reducing the risk of diabetes complications. However, few studies have systematically examined whether DSME follows AADE 7 Self-Care Behaviors (AADE7™). Therefore, this study aimed to investigate the control effect of AADE7™-based management on laboratory test indicators of diabetic patients.

**Methods:**

The patients with diabetes who received shared care management in our hospital between June 2014 and April 2022 were analyzed retrospectively. According to the process of outpatient consultation, each patient received health education provided by diabetes education nurses and dietitians after consultation. Health education was a process from assessment to health guidance. The basic information of all patients was recorded, and AADE7™ behavior assessment and health education session were conducted through interviews. A total of 13,650 were given shared care management, requiring more than 6 follow-up visits per year, as well as health education. It was reassessed annually according to AADE standards. The impact of the patients' behavior change after the AADE7™-based management on the relevant test indicators was observed.

**Results:**

After eight years of intervention, a total of 8319 samples were obtained after excluding the outliers. Stepwise regression analysis was performed, and the results showed that, with other conditions held constant, a greater number of days per week to follow a healthy diet, to take hypoglycemic medication as prescribed, to monitor blood glucose, and to exercise and higher education level were associated with lower levels of glycosylated hemoglobin. The change from drinking to nondrinking was associated with lower triglycerides. If low blood glucose was monitored, patients who reviewed and took immediate action showed lower levels of low-density lipoprotein, urine microalbumin, and urine microalbumin/creatinine ratio compared with those without review and immediate action. Significance tests for each term showed *P* value <0.05.

**Conclusions:**

The AADE7™ framework is a tool supporting patient-centered self-management and education. In the AADE7™ standards, successful self-management is considered as a key outcome in the care of patients with diabetes and related diseases. This tool can effectively improve patient compliance and increase the rate of blood glucose compliance rates in patients with diabetes and therefore is worthy of clinical promotion.

## 1. Introduction

The prevalence of diabetes has increased significantly in China over the past 30 years, reaching 11.2% in 2015–2017. However, despite the improvement made in the rates of diabetes awareness (36.5%), treatment (32.2%), and control (49.2%), they are still at a low level [1]. Patients with diabetes often develop metabolic syndrome simultaneously. The control of the patient's blood pressure, lipid, blood glucose, and weight control should be based on improving lifestyle and supplemented by rational use of medication according to the patient's condition. Glycosylated hemoglobin (HbA1c) is the main indicator to reflect blood glucose control. Crucially, the control goal should follow the principle of individualization; that is, patients are managed in a stratified manner according to their age, disease duration, medication use, health status, and other factors [2, 3]. The American Diabetes Association (ADA)'s Standards of Medical Care in Diabetes-2017 for diabetes calls for “a patient-centered communication style that uses active listening, elicits patient preferences and beliefs, and assesses literacy, numeracy, and potential barriers to care” to “optimize patient health outcomes and health-related quality of life.” In 1997, the Centers for Medicare and Medicaid Services (CMS) challenged the American Association of Diabetes Educators (AADE) to identify the unique outcome measures of diabetes self-management education (DESM) [4]. AADE convened a task force to determine what to measure, when to monitor, and how to manage the chronic conditions associated with diabetes education and care [5]. The task force defined the unique outcomes of DESM as “behavior change” and seven self-care behaviors that can promote successful and effective diabetes self-management, called AADE 7 Self-Care Behaviors (AADE7™). The work of the initial task force included mapping the 15 original content areas of the 1995 National Standards for Diabetes Self-Management Education Programs, obtaining outcomes through the development and testing of diabetes education evaluation tools [6] and reaching consensus on DSME outcome measures using AADE7™ [7]. AADE7™ provides a framework that shifts the focus from an educational content-driven practice to an outcome-driven practice using person-centered and self-defined goals [8, 9]. Effective diabetes education needs to go beyond knowledge transfer; it needs to manage and support behavior changes and influence clinical and health-related outcomes. Therefore, this paper examined the impact of AADE7™-based assessment on patients' behaviors and blood glucose compliance rates.

## 2. Study Subjects and Methods

### 2.1. Study Subjects

A total of 13,650 patients with diabetes who received share care in our hospital from June 2014 to April 2022 were selected. Before data analysis, outliers were excluded, and finally, 8,319 samples were obtained. Empty items or logical errors in patient data were excluded. This paper examines the relationship between following the AADE7™ practice standards on the effectiveness of test index control in patients with diabetes. This study was approved by the Ethics Committee of Beijing Ruijing Diabetes Hospital (2023001).

Inclusion criteria were as follows: ① a diagnosis of diabetes meeting the World Health Organization diagnostic criteria for diabetes; ② no communication barriers; ③ willing to and receiving behavior management for diabetes and voluntarily signing the informed consent form. Exclusion criteria were as follows: ① communication disability; ② disability to receive health education telephone follow-up for other reasons.

### 2.2. Methods

Our hospital has applied the shared care model for managing diabetic patients since 2014. Shared care was a model with joint participation of physicians, dietitians, diabetes education primary nurses, and exercise instructors. They worked in cooperation to provide diagnosis and treatment, health education, and follow-up in order to enhance the self-management ability of diabetic patients, improve their blood glucose compliance rate, and reduce their occurrence and development of complications. Self-management is an important concept to emphasize, because diabetic patients have to make and execute choices which will regularly and cyclically affect the patient's health. Effective self-management is a process that involves learning about disease-related knowledge, identifying personal goals, weighing the pros and cons of various treatment recommendations, making insightful choices, improving one's emotional and behavioral skills to support those choices, and evaluating one's effectiveness in reaching self-planned goals.

Diabetes educators are an essential part of a diabetes care team. Diabetes educators understand the impact of personal health behaviors and lifestyle on disease, and such understanding is necessary for a comprehensive treatment program and DESM. Therefore, our hospital followed the AADE7™ standards for assessing patient behaviors, which include healthy eating, being physically active, blood glucose monitoring, taking medication, problem solving, healthy coping, and reducing risks. The number of days in a week that patients complied with each of the seven behaviors was recorded. Diabetes education nurses conducted targeted health education according to the evaluation results of patients, including healthy diet, exercise, high and low blood sugar management, blood sugar monitoring, oral medication, reducing risk factors, and other aspects of health education. Each health education session lasts 60 minutes. Depending on the patient's situation, we assist the patient to set goals and conduct regular follow-ups, with more than 6 follow-up visits per year, in order to better help the patient maintain healthy behavior.

### 2.3. Observation Indicators

After the patients were managed using the AADE7™ standards, the relationship between patients' behaviors and laboratory test indicators was assessed. The behaviors included drinking status, education level, number of days per week to follow a healthy diet, number of days per week to exercise, number of days per week to monitor blood glucose, number of days per week to take hypoglycemic medication as prescribed, reviewing and taking immediate action when high blood glucose was monitored, and reviewing and taking immediate action when low blood glucose was monitored. The indicators were HbA1c, triglycerides (TG), low-density lipoprotein (LDL), urine microalbumin, and urine microalbumin/creatinine ratio.

### 2.4. Statistical Methods

Descriptive statistical analysis of variables was performed by SPSS 26.0. A multiple linear regression was used to describe the relationship between variables by fitting a line. The parameter estimates were obtained using the least squares method, and the test of goodness of fit (coefficient of determination), the significance test of the regression equation (*F*-test), and the significance test of the regression parameters (*t*-test) were performed. One of the prerequisite assumptions of multiple linear regression is that there is a linear relationship between the dependent variable *y* and the independent variables *x*1, *x*2, *x*3,…, *xk*. Therefore, to test this assumption, correlation analysis between the variables was carried out using EViews11. The Pearson correlation coefficient was used since the variables were all continuous variables.

## 3. Results

### 3.1. Analysis of Each Variable in Diabetic Patients

As shown in Table 1, HbA1c, TG, LDL, urine microalbumin, and urine microalbumin/creatinine ratio served as the dependent variables. Then, the following variable were included in the model: drinking status, education level, number of days per week to follow a healthy diet, number of days per week to exercise, number of days per week to monitor blood glucose, number of days per week to take hypoglycemic medication as prescribed, reviewing and taking immediate action when high blood glucose was monitored, and reviewing and taking immediate action when low blood glucose was monitored. A multiple linear regression model was used to explore the relationship between the variables.

### 3.2. Influencing Factors of Glycosylated Hemoglobin in Diabetic Patients

After stepwise regression procedures with HbA1c as the dependent variable, a total of five influencing factors were screened out, and all had negative influence coefficients. This result indicates that with other things held constant, a greater number of days per week to follow a healthy diet, to take hypoglycemic medication as prescribed, to monitor blood glucose, and to exercise and higher education level were associated with lower HbA1c values (Table 2).

### 3.3. Influencing Factors of Triglycerides in Diabetic Patients

After stepwise regression procedures triglycerides as the dependent variable, a total of five influencing factors were screened out. The specific results demonstrated that a greater number of days per week to follow a healthy diet, to exercise, and to monitor blood glucose were associated with lower TG levels. The higher the education level, the higher the TG levels. In addition, TG values decreased by −0.118 units for each level of change in drinking status (from drinking to nondrinking) (Table 3).

### 3.4. Influencing Factors of Low-Density Lipoprotein in Diabetic Patients

Stepwise regression analysis with LDL as the dependent variable screened out five influencing factors. Specifically, a greater number of days per week to follow a healthy diet, to take hypoglycemic medication as prescribed, to monitor blood glucose, and to exercise were associated with lower LDL levels. Compared to those who reviewed and took immediate action when their blood glucose was monitored low, those without review and immediate action had an average increase of 0.084 units of LDL values (Table 4).

### 3.5. Influencing Factors of Urine Microalbumin in Diabetic Patients

With urine microalbumin as the dependent variable, a total of three influencing factors were identified after stepwise regression. The results showed that urine microalbumin was lower in patients with higher education level and a greater number of days per week to exercise. In addition, compared to those who reviewed and took immediate action when their blood glucose was monitored low, those without review and immediate action had an average decrease of 15.003 units of urine microalbumin (Table 5).

### 3.6. Influencing Factors of Urine Microalbumin/Creatinine Ratio in Diabetic Patients

Stepwise regression with a urine microalbumin/creatinine ratio as the dependent variable identified only one influencing factor (education level). With all other things held constant, higher education level was associated with a lower urine microalbumin/creatinine ratio (Table 6).

## 4. Discussion

With HbA1c as the dependent variable, five influencing factors were screened out; with other things held constant, lower HbA1c was associated with a greater number of days per week to follow a healthy diet, to take hypoglycemic medication as prescribed, to monitor blood glucose, to exercise, and higher education level. This result fully supports that lifestyle interventions can effectively control blood glucose in diabetic patients [3]. HbA1c is the current “gold standard” for evaluating the glycemic control of diabetic patients, and it is of great value in the management of diabetes [10]. HbA1c control goals should follow the patient-centered and personalized principle; that is, stratified management is performed according to patients' age, disease duration, health status, risk of adverse drug reactions, and other factors [3]. The patients in this study were managed based on the AADE7™ standards; the diabetes education nurses assessed the basic patient profile during the health education session and then helped patients set individualized goals. Subjects enrolled in the UK Prospective Diabetes Study were newly diagnosed T2DM patients [11]. The results showed that the intensive blood glucose lowering group with HbA1c reduced to less than 7.0% had a significant microvascular benefit although there was no significant macrovascular benefit [11]. A 10-year follow-up study also found a marked reduction in the risk of macrovascular disease, microvascular disease, and all-cause mortality in the intensive blood glucose control group, which is known as the benign “metabolic memory” effect [12]. Various studies have confirmed the feasibility of this AADE7™-based management model for diabetes.

In this study, TG and LDL were used as dependent variables and were influenced by the number of days per week of healthy eating, the number of days per week of exercise, and the number of days per week of blood glucose monitoring. In addition, drinking status and education level also influence TG levels, and reviewing and taking immediate action when low blood glucose was monitored and the number of days per week of taking hypoglycemic medication as prescribed were factors affecting LDL. The results confirm that behavioral changes in patients with diabetes are effective in improving their lipid. In Chinese guidelines for type 2 diabetes, patients with type 2 diabetes, patients with long duration of diabetes, old age, and cardiovascular disease are recommended to continue to receive comprehensive management such as glucose lowering, blood pressure lowering, and lipid regulation (mainly LDL cholesterol lowering) [3]. These management measures aim to reduce the risk of cardiovascular events, progression of microvascular complications, and death [3]. The present study is consistent with the findings of Li et al. A shared care model by Li et al. achieved effective manage patient management, enhancement of patients' self-management, and rational and standardized application of glucose-lowering medication [13]; consequently, a glucose compliance rate could be gradually improved. Patients with higher education level have better ability to accept diabetes education and better execution, showing better control of lipid.

Meanwhile, in this study, the dependent variable urine microalbumin was influenced by patients' education level, number of days per week to exercise, and reviewing and taking immediate action when low blood glucose was monitored. The dependent variable urine microalbumin/creatinine ratio was only affected by education level, suggesting that higher education level was associated with a lower urine microalbumin/creatinine ratio. The National Kidney Foundation Kidney Disease Outcomes Quality Initiative recommends the use of the urine microalbumin/creatinine ratio to assess urinary albumin excretion in patients with kidney disease [14]. Diabetic nephropathy is a common microvascular complication of diabetes. Urine microalbumin and urine microalbumin/creatinine ratios can better evaluate the severity of kidney disease in diabetic patients [3]. In this study, only the urinary microalbumin/creatinine ratio was captured for correlation analysis, and eGFR numerical calculation was not performed. Analysis and discovery evaluation of patients through AADE7™, individualized health education, behavioral goals, and long-term follow-up management can effectively control urinary microalbumin/creatinine ratios and prevent and delay the occurrence of complications.

## 5. Conclusion

This study truly describes the impact of patient behavior change on laboratory test indicators since the implementation of a shared care model in our hospital. AADE7™ used in the shared care system can provide qualitative or quantitative assessment for each patient's condition, facilitating effective health education for patients by diabetes educators and achieving initial success after long-term management. Therefore, it is worthy of clinical promotion.

## Figures and Tables

**Table 1:** Analysis of each variable in diabetic patients.

Variables	Sample size	Maximum value	Minimum value	Average value	Standard deviation	Median
Glycosylated hemoglobin	8319	19	4.1	8.572	2.044	8.2
Triglycerides	8319	31.14	0.22	2.124	1.84	1.65
High-density lipoprotein	8319	5.93	0.17	1.385	0.351	1.35
Low-density lipoprotein	8319	8.89	−1.23	2.776	0.904	2.73
Urine microalbumin	8319	6636.19	0.01	68.066	189.61	15.03
Urine microalbumin/creatinine ratio	8319	26072.1	0.02	79.774	387.924	13.49
Drinking status	8319	3	1	2.696	0.71	3
Education level	8319	5	1	3.423	0.833	3
Healthy eating (day/week)	8319	7	0	2.963	2.825	3
Exercise (day/week)	8319	7	0	4.733	2.923	7
Blood glucose monitoring (day/week)	8319	7	0	2.089	2.278	2
Taking hypoglycemic medication as prescribed (day/week)	8319	7	0	5.097	2.992	7
Reviewing and taking immediate action when high blood glucose was monitored	8319	2	1	1.529	0.499	2
Reviewing and taking immediate action when low blood glucose was monitored	8319	2	1	1.447	0.497	1

**Table 2:** HbA1c in diabetic patients influencing factors.

	Coef.	se	Std. coef	*T*	*P*	VIF
Constants	10.094	0.108	0	93.067	<0.001	—
Healthy eating according to day/week	−0.085	0.008	−0.118	−10.166	<0.001	1.186
Take glucose-lowering medication as prescribed (daily/weekly)	−0.068	0.007	−0.099	−9.027	<0.001	1.059
Blood glucose monitoring (daily/weekly)	−0.077	0.01	−0.086	−7.565	<0.001	1.127
Education level	−0.177	0.026	−0.072	−6.742	<0.001	1.007
Exercise (day/week)	−0.033	0.008	−0.048	−4.283	<0.001	1.095

*Note*. HbA1c, glycosylated hemoglobin; ^*∗∗∗*^, ^*∗∗*^, and ^*∗*^1%, 5%, and 10% significance levels, respectively.

**Table 3:** Factors influencing triglycerides in diabetic patients.

	Coef.	Se	Std. coef	*t*	*P*	VIF
Constants	2.651	0.123	0	21.528	<0.001	—
Healthy eating according to day/week	−0.046	0.008	−0.07	−5.995	<0.001	1.168
Exercise (day/week)	−0.044	0.007	−0.07	−6.178	<0.001	1.095
Alcohol consumption status	−0.118	0.028	−0.046	−4.189	<0.001	1.01
Blood glucose monitoring (daily/weekly)	−0.029	0.009	−0.036	−3.152	0.002	1.109
Education level	0.058	0.024	0.026	2.406	0.016	1.007

*Note*. ^*∗∗∗*^, ^*∗∗*^, and ^*∗*^1%, 5%, and 10% significance levels, respectively.

**Table 4:** Influencing factors of low-density lipoprotein in diabetic patients.

	Nonstandardized coefficient		Standardized coefficient	*t*	*P*	VIF	*R* ^2^	Adjusted *R*^2^	*F*
	*B*	Standard error	Beta						
Constants	2.828	0.046	0	61.294	0.000^*∗∗∗*^	—	0.015	0.014	*F* = 24.941, *P*=0.000^*∗∗∗*^
Healthy eating (day/week)	−0.017	0.004	−0.053	−4.32	0.000^*∗∗∗*^	1.282			
Reviewing and taking immediate action when low blood glucose was monitored	0.084	0.022	0.046	3.854	0.000^*∗∗∗*^	1.204			
Exercise (day/week)	−0.014	0.004	−0.047	−4.09	0.000^*∗∗∗*^	1.094			
Taking hypoglycemic medication as prescribed (day/week)	−0.007	0.003	−0.023	−2.032	0.042^*∗∗*^	1.078			
Blood glucose monitoring (day/week)	−0.009	0.005	−0.023	−1.969	0.049^*∗∗*^	1.15			
Dependent variable: low-density lipoprotein									

*Note*. ^*∗∗∗*^, ^*∗∗*^, and ^*∗*^1%, 5%, and 10% significance levels, respectively.

**Table 5:** Influencing factors of urine microalbumin in diabetic patients.

	Nonstandardized coefficient		Standardized coefficient	*t*	*P*	VIF	*R* ^2^	Adjusted *R*^2^	*F*
	*B*	Standard error	Beta						
Constants	148.381	11.441	0	12.969	0.000^*∗∗∗*^	—	0.006	0.006	*F* = 17.17, *P*=0.000^*∗∗∗*^
Education level	−14.687	2.492	−0.064	−5.895	0.000^*∗∗∗*^	1.002			
Reviewing and taking immediate action when low blood glucose was monitored	−15.003	4.206	−0.039	−3.567	0.000^*∗∗∗*^	1.018			
Exercise (day/week)	−1.761	0.716	−0.027	−2.46	0.014^*∗∗*^	1.019			
Dependent variable: urine microalbumin									

*Note*. ^*∗∗∗*^, ^*∗∗*^, and ^*∗*^1%, 5%, and 10% significance levels, respectively.

**Table 6:** Factors influencing the change in the urine microalbumin/creatinine ratio in diabetic patients.

	Nonstandardized coefficient		Standardized coefficient	*t*	*P*	VIF	*R* ^2^	Adjusted *R*^2^	*F*
	*B*	Standard error	Beta						
Constants	157.976	17.975	0	8.789	0.000^*∗∗∗*^	—	0.002	0.002	*F* = 20.048, *P*=0.000^*∗∗∗*^
Education level	−22.848	5.103	−0.049	−4.478	0.000^*∗∗∗*^	1			
Dependent variable: urine microalbumin/creatinine ratio									

*Note*. ^*∗∗∗*^, ^*∗∗*^, and ^*∗*^1%, 5%, and 10% significance levels, respectively.

## Data Availability

The data used to support the findings of this study are available from the corresponding author upon request.
